# Identifying acute kidney injury in children: comparing electronic alerts with health record data

**DOI:** 10.1186/s12882-025-03961-3

**Published:** 2025-02-13

**Authors:** Lucy Plumb, Manuela Savino, Anna Casula, Manish D. Sinha, Carol D. Inward, Stephen D. Marks, James Medcalf, Dorothea Nitsch

**Affiliations:** 1 UK Renal Registry, UK Kidney Association, Building 20A1, Filton 20, Filton, Bristol, BS34 7RR UK; 2 Population Health Sciences, University of Bristol Medical School, Oakfield Grove, Bristol, BS8 2BN UK; 3 Bristol Royal Infirmary, University Hospitals Bristol and Weston NHS Foundation Trust, Bristol, UK; 4 Evelina London Children’s Hospital, Guys and St Thomas’ NHS Foundation Trust, London, UK; 5 British Heart Foundation Centre, Kings College London, London, UK; 6 Department of Paediatric Nephrology, University Hospitals Bristol & Weston NHS Foundation Trust, Bristol, UK; 7 Department of Paediatric Nephrology, Great Ormond Street Hospital for Children NHS Foundation Trust, London, UK; 8 NIHR Great Ormond Street Hospital Biomedical Research Centre, University College London Great Ormond Street Institute of Child Health, London, UK; 9 Depratment of Cardiovascular Sciences, University of Leicester, Leicester, UK; 10 Leicester General Hospital, Leicester, UK; 11 Department of Non-Communicable Disease Epidemiology, London School of Hygiene and Tropical Medicine, London, UK

**Keywords:** Pediatrics, Child, Acute Kidney Injury, Electronic health records, Epidemiology

## Abstract

**Background:**

Electronic (e-)alerts for rising serum creatinine values are increasingly used as clinical indicators of acute kidney injury (AKI). The aim of this study was to investigate to what degree AKI episodes, as identified using e-alerts, correlated with coding for AKI in the hospital record for a national cohort of hospitalised children and examine whether coding corresponded with 30-day mortality after an AKI episode.

**Methods:**

A cross-section of AKI episodes based on alerts issued for children under 18 years in England during 2017 were linked to hospital records. Multivariable logistic regression was used to examine patient and clinical factors associated with AKI coding. Agreement between coding and 30-day mortality was examined at hospital level.

**Results:**

6272 AKI episodes in 5582 hospitalised children were analysed. Overall, coding was poor (19.7%). Older age, living in the least deprived quintile (odds ratio (OR) 1.4, 95% Confidence Interval (CI) 1.1, 1.7) and higher peak AKI stage (stage 1 reference; stage 2 OR 2.0, 95% CI 1.7, 2.4; stage 3 OR 8.6, 95% CI 7.1, 10.6) were associated with higher likelihood of coding in the hospital record. AKI episodes during birth admissions were less likely to be coded (OR 0.4, 95% CI 0.3, 0.5). No correlation was seen between coding and 30-day mortality.

**Conclusions:**

The proportion of AKI alert-identified episodes coded in the hospital record is low, suggesting under-recognition and underestimation of AKI incidence. Understanding the reasons for inequalities in coding, variation in coding between hospitals and how alerts can enhance clinical recognition is needed.

**Supplementary Information:**

The online version contains supplementary material available at 10.1186/s12882-025-03961-3.

## Introduction

Acute Kidney Injury (AKI) is associated with adverse outcomes in children including increased length of hospitalisation and risk of death and confers a longer-term risk of kidney impairment. There is some evidence that the incidence of AKI is increasing among adult patients, which may in part be due to increased recognition, but also concurrent increases in rates of sepsis, major surgery, and co-existing disease [1]. In children, temporal trends are less well reported. In the UK, the prevalence of AKI documented within the primary care record was noted to rise over a 16-year period [2]. Robust AKI surveillance is crucial to monitoring trends, identifying risk factors and adverse outcomes, and implementing high quality care, which in turn is dependent on its recognition. To facilitate timely detection, in 2014, National Health Service (NHS) England implemented a patient safety electronic (e-) alert, requiring all NHS trusts to flag abnormal or rising serum creatinine values as suggestive of AKI, using an algorithm that aligns with the internationally accepted definition for detection and staging of AKI [3, 4]. Automated e-alerts appear contemporaneously alongside creatinine results in reporting systems and are available to clinicians. In England, these data are sent to the UK Renal Registry (UKRR) for reporting and analysis [5].

Broadly speaking, much epidemiological AKI research has relied on disease surveillance through electronic health record coding. The introduction of the national AKI alert warning system offers an opportunity to describe and compare AKI identified among children using two different methodologies. To what degree AKI episodes defined using e-alerts correlate with coding in the clinical record is unknown for children. Understanding how e-alerts correlate with clinical recognition and documentation within the electronic health record is necessary to understand how both methods may facilitate surveillance of AKI and its outcomes. Our aim was to determine what proportion of AKI episodes, identified using the AKI e-alert system established in NHS laboratories across England, were coded within hospital records, and to determine patient and clinical factors associated with coding. A secondary objective was to determine whether AKI coding within the electronic hospital record, as a proxy for clinical recognition, corresponded with 30-day mortality at hospital level.

## Methods

A cross-sectional study was conducted to address the study aims. The study population comprised children under 18 years of age in England who experienced an AKI episode, as identified by e-alerts, between January 1, 2017, and December 31, 2017, that was associated with a hospital admission (AKI-associated hospitalisation) in whom linkage to the electronic hospital record for England, Hospital Episode Statistics (HES), was available. AKI alerts per patient were amalgamated into episodes as has been described previously [6, 7] and comprised all e-alerts occurring without a 30-day interval, following which time a separate, subsequent episode was considered to have occurred. The algorithm used by NHS laboratories in England, which was developed by the ‘Think Kidneys’ working group and aligns with KDIGO guidance for AKI detection, requires a baseline serum creatinine value to trigger an e-alert. If a baseline value was available in the previous seven days, the lowest available value was used for reference; if older serum creatinine values were available (8–365 days prior to testing), the median of these results was used [4]. Children with more than one episode of AKI during this period could be included multiple times. Children receiving long-term kidney replacement therapy (KRT) during the period were identified and excluded. Laboratories which did not submit data in time for HES linkage or AKI-episodes that started before 2017 were excluded from analysis. Due to physiological changes in serum creatinine after birth, episodes occurring solely within the first 3 days of life were also excluded; alerts that continued to flag beyond this timeframe were included.

AKI was categorised as community acquired (CA) if the episode started prior to, or within the first 2 days of a hospital admission, and hospital-acquired (HA) if an AKI episode occurred from day 3 of a hospitalisation. Exceptions to this rule were children that experienced an AKI episode during their birth admission, who were referred to separately as the ‘birth cohort’. Children in the birth cohort were further stratified by whether they were coded as being premature or not, based on the presence of a relevant ICD-10 code in their hospital record (ICD-10 codes P072-P073, H351).

AKI e-alert data is sent to the UKRR for reporting and analysis, along with a Master Patient Index (MPI) of data which includes date of birth of the patient, sex, residential postcode, and creatinine value triggering alert. Age and sex were obtained from the AKI-MPI. Area-level deprivation quintiles based on the Index of Multiple Deprivation for England were assigned using residential postcode [8]. Ethnicity was derived from HES-linked data. Peak AKI was defined by the highest AKI alert stage reached within 30-days of the start of the episode.

The outcome of interest was the sensitivity of hospital records for recording an AKI episode, determined using AKI e-alerts: this was defined as the presence of an International Classification of Diseases, tenth revision (ICD-10) code for AKI (N17.x, yes/no) in any diagnosis field contained within the electronic hospital record during the associated hospital admission. Within the NHS, clinical coding is the translation of the patient record into coded data, capturing information about diagnoses, health-related conditions and the care provided, which is performed by clinical coders accredited to a national standard [9]. A second outcome of death within 30-days of an AKI episode was also examined, captured using the NHS Demographics Batch Service.

Characteristics of the AKI episodes included in the analysis, overall and stratified by peak AKI stage, are presented using basic descriptive statistics; admission to critical care is also reported. Use of KRT during the associated hospitalisation was determined by the presence of an Office of Population Censuses and Surveys Classification of Interventions and Procedures (OPCS) code in the hospital record (additional Table 1). Funnel plots were used to highlight age-adjusted proportions of AKI episodes coded within the electronic hospital record for each hospital submitting data, stratified by peak AKI stage. Univariable and multivariable (age- and sex-adjusted) logistic regression analyses were performed to investigate demographic and clinical variables predictive of AKI coding. Analyses were multi-level, stratified by hospital; hospitals reporting ≤ 10 episodes of AKI were retained in the analysis but grouped as one stratum. Age-adjusted scatterplots were used to examine the association between N17 coding in HES and 30-day mortality from AKI episode start, for the total cohort and excluding the birth cohort. All analyses were performed using SAS, version 9.4.

The UK Renal Registry collects, processes and shares patient-level data without individual patient consent for audit and research purposes under Sect. 251 of the NHS Act (2006) granted by the Health Research Authority’s Confidentiality Advisory Group (reference: 16/CAG/0153) and therefore consent to participate was not obtained.

## Results

The UKRR received data on 6272 hospitalisations associated with an AKI e-alert episode that occurred in 5582 children in 2017. Of these, 732 (11.7%) hospitalisations were associated with a birth admission and 46.8% occurred either prior to, or within 2 days of admission. Table 1 outlines the demographic and clinical characteristics for each episode included in the analysis, overall and stratified by peak AKI stage. Most children experienced AKI stage 1 at start (79.5%) and at peak (65.8%); AKI stage 3 was seen in 6.6% at start and 13.1% at peak. Over one-fifth of the study cohort was admitted to critical care during their AKI-associated hospitalisation (*n* = 1402, 22.4%), with higher proportions of critical care admission seen with higher peak AKI stage. KRT use was recorded in 0.9%, 1.5% and 10.3% of children who experienced peak AKI stages 1, 2 and 3, respectively. Most children in the birth cohort who experienced an AKI were premature (*n* = 519, 70.9%). Episodes predominantly came from hospitals which may have been providing secondary or specialist care (82.6%); 17.4% of episodes came from hospital trusts which provide paediatric-specific care (list of laboratories submitting data available in additional Table 2).

**Table 1:** Description of AKI alert-associated hospitalisations included in analysis (*n* = 6272)

	**All episodes** **N (%)**	**Episodes by peak AKI stage (%)**		
		**1**	**2**	**3**
N	6272	4128	1321	823
**Male sex**	3309 (52.8)	52.33	52.69	55.04
**Age group (years)**				
1	1682 (26.8)	23.1	34.5	32.9
1- < 2	641 (10.2)	10.1	10.8	9.8
2- < 4	797 (12.7)	13.7	10.5	11.4
4- < 8	1090 (17.4)	18.4	16.4	13.7
8- < 12	636 (10.1)	10.4	10.1	9.1
12- < 16	753 (12.0)	12.6	10.1	12.4
16- < 18	673 (10.7)	11.7	7.7	10.6
**Ethnicity**				
Asian	556 (9.1)	8.8	8.9	10.5
Black	330 (5.4)	5.4	5.3	5.5
Other	621 (10.1)	10.2	10.3	9.5
White	4627 (75.4)	75.6	75.4	74.5
Missing	138 (2.2)	2.2	1.7	2.9
**Deprivation quintile**				
1*-least deprived*	814 (13.1)	13.4	12.6	12.0
2	1057 (16.9)	17.1	16.5	17.0
3	1142 (18.3)	18.0	18.5	19.4
4	1366 (21.9)	21.8	21.2	23.6
5*-most deprived*	1859 (29.8)	29.7	31.2	28.1
Missing	34 (0.5)	0.5	0.6	0.5
**AKI stage at start**				
1	4989 (79.5)	100	44.7	32.9
2	869 (13.9)	0	55.3	16.8
3	414 (6.6)	0	0	50.3
**Peak AKI stage**				
1	4128 (65.8)	100		
2	1321 (21.1)		100	
3	823 (13.1)			100
**AKI admission type**				
Birth cohort	732 (11.7)	9.3	15.6	17.3
Of which preterm infants	519 (70.9)	72.4	70.0	68.3
Hospital acquired (excl. birth)	2608 (41.6)	42.6	42.5	35.2
Community acquired-admitted	2932 (46.8)	48.1	41.9	47.5
**Source of alert**				
District hospital	5182 (82.6)	83.4	82.0	79.7
Paeds hospital^a^	1090 (17.4)	16.6	18.0	20.3
Admission to critical care^b^	1402 (22.4)	18.8	27.6	31.4
**Proportion coded for AKI in hospital record**	1236 (19.7)	14.5	22.3	41.4

^a^*n* = *3. *Abbreviations: *AKI* Acute kidney injury. ^b^At any point during the AKI episode (e.g., critical care admission at AKI start, AKI start in critical care, AKI start then admission to critical care)

**Table 2:** Univariable and multivariable (age- and sex-adjusted) associations with AKI coding in hospital record, with analysis stratified by hospital

	**Univariable OR (95% CI)**	**Adjusted OR (95% CI)**
**Male sex**	0.9 (0.8, 1.1)	1.0 (0.9, 1.1)
Female	Reference	Reference
**Age group (years)**		
2	Reference	Reference
2- < 4	1.3 (1.0, 1.6)	1.3 (1.0, 1.6)
4- < 8	1.3 (1.1, 1.7)	1.3 (1.1, 1.7)
8- < 12	1.9 (1.5, 2.4)	1.9 (1.5, 2.4)
12- < 16	2.2 (1.7, 2.7)	2.2 (1.7, 2.7)
16- < 18	2.6 (2.1, 3.4)	2.6 (2.1, 3.4)
**Ethnicity**		
White	Reference	Reference
South Asian	1.0 (0.8, 1.3)	1.0 (0.8, 1.3)
Black	1.2 (0.9, 1.7)	1.2 (0.8, 1.7)
Other	1.0 (0.8, 1.3)	1.0 (0.8, 1.3)
**Area-level deprivation**		
1*-least deprived*	1.4 (1.1, 1.8)	1.4 (1.1, 1.7)
2	1.0 (0.8, 1.3)	1.0 (0.8, 1.3)
3	1.3 (1.0, 1.6)	1.3 (1.0, 1.6)
4	1.1 (0.9, 1.3)	1.1 (0.9, 1.3)
5*-most deprived*	Reference	Reference
**Birth cohort**	0.3 (0.2, 0.4)	0.4 (0.3, 0.5)
Non-birth cohort	Reference	Reference
**Community acquired-admitted**	1.0 (0.8, 1.2)	1.0 (0.8, 1.2)
Hospital acquired^a^	Reference	Reference
**Birth cohort**	0.3 (0.2, 0.4)	0.4 (0.3, 0.5)
Hospital acquired	Reference	Reference
**Community acquired-admitted**	1.0 (0.8, 1.1)	1.0 (0.8, 1.1)
Hospital acquired	Reference	Reference
**Peak AKI stage**		
Stage 1	Reference	Reference
Stage 2	1.9 (1.6, 2.3)	2.0 (1.7, 2.4)
Stage 3	8.1 (6.7, 9.9)	8.6 (7.1, 10.6)

^a^Birth cohort excluded

When stratified by peak AKI stage, similar trends in sex and deprivation were noted. Higher proportions of children aged < 2 years were seen for peak stages 2 and 3 compared with 1, predominantly driven by children < 1 year of age; higher proportions of children from the birth cohort were also noted with higher peak AKI stage. A higher proportion of Asian children were noted to experience AKI peak stage 3 compared to lower stages. Additionally, episodes from paediatric hospital trusts contributed more to the higher peak AKI stages.

**Fig. 1:**
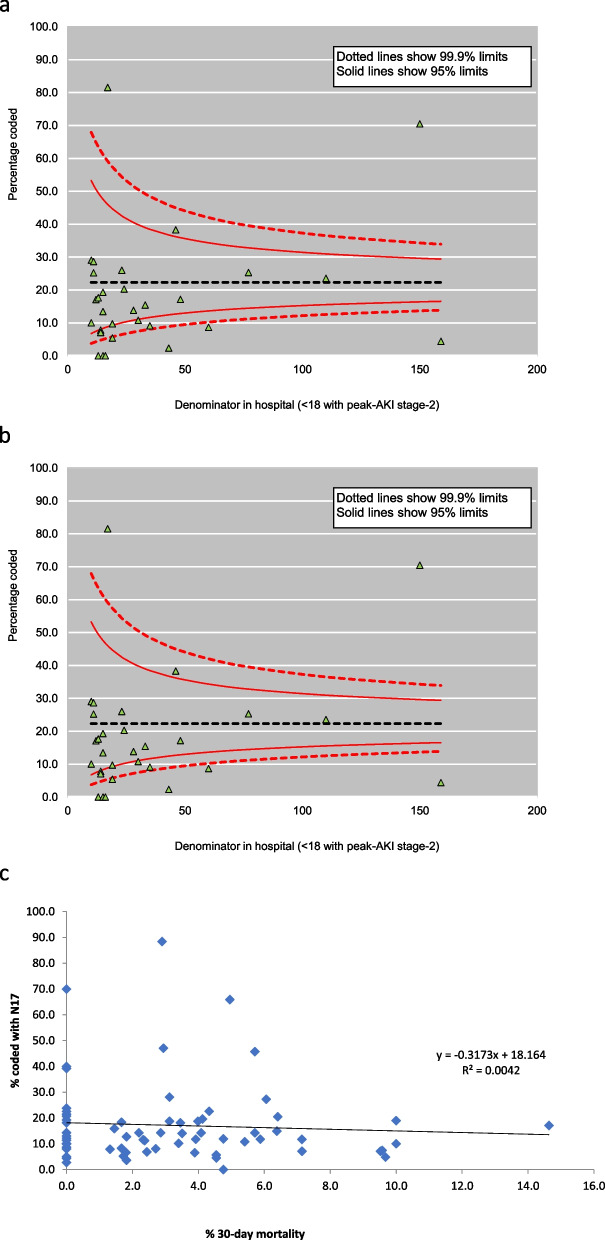
Age-adjusted percentage of patients with peak AKI stage **a**) one **b**) two **c**) three during hospitalised episode coded within electronic record, by hospital

Overall, only 19.7% of AKI-episodes identified using e-alerts were coded, with the proportion of episodes coded increasing with peak AKI stage: 14.5%, 22.3% and 41.4% of episodes coded with peak AKI stages 1, 2 and 3, respectively. A similar pattern was noted at hospital level: excluding hospitals with few (≤ 10) AKI cases, the age-adjusted median percentage of coded AKI episodes was 7.2% (Interquartile range, IQR, 3.4–12.6%) for stage 1, 14.6% (IQR 8.0–24.8%) for stage 2 and 30.2% (IQR 19.8–43.1%) for stage 3 (Figs. 1a-c). In the birth cohort, 9% of children were coded as having AKI; compared to preterm infants, a higher proportion of non-preterm infants were coded for AKI, ranging from 9.4% for peak AKI stage 1, to 28.9% for peak AKI stage 3 (additional Table 3).

Demographic and clinical variables were associated higher likelihood of coding of AKI e-alert episodes (Table 2). In both univariable and age- and sex-adjusted models, higher odds of coding within the hospital record were noted for older age-groups: compared to children under 2 years, children aged 2–4 years had 28% higher odds (95% confidence interval, CI 1.00, 1.63) of being coded, while children aged 16–18 years had 2.6 times higher odds (95% 2.06, 3.35) of coding. Similar findings were noted when models were stratified by peak AKI severity (stage 1 and stages 2/3 combined), although effect estimates were attenuated. There was a weak association between area-level socioeconomic deprivation and coding: compared to children living in the most deprived quintile, children in less deprived quintiles 1 and 3 had higher odds of coding, although for quintile 2 this estimate was consistent with chance. No association was seen by ethnicity. Compared to hospital-acquired episodes, children experiencing AKI during their birth admission had 63% lower odds of the episode being coded. No differences were noted between community-acquired hospitalised episodes compared to those with hospital-acquired AKI. Likelihood of coding was also higher with peak AKI stage, with children experiencing peak stage 3 over 8 times more likely to be coded for AKI compared to those with stage 1. Table 2 Univariable and multivariable (age- and sex-adjusted) associations with AKI coding in hospital record, with analysis stratified by hospital.

**Fig. 2:**
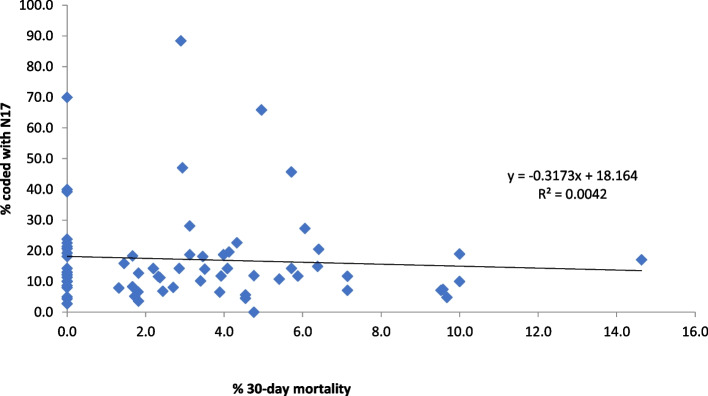
Correlation between N17 coding and 30-day mortality, by hospital

At hospital level, no correlation was noted between proportion of AKI e-alert episodes coded and 30-day mortality for children in England (Fig. 2); similar findings were noted when the birth cohort was excluded from analysis (R^2^ 0.001).

Several post hoc analyses were performed. We examined critical care admission, KRT use, and the median length of stay by AKI coding status to determine whether differences were present. In general, higher proportions of AKI episodes associated with critical care admission or KRT use were coded in the hospital record compared to those without (additional tables 4 and 5). For both outcomes however, the main differences in coding appeared to be driven by peak AKI stage. On a hospital level, no correlation was seen for length of stay by AKI-coding status. At patient level, length of stay was generally longer when patients were coded for AKI during their associated hospital stay and when stratified by peak AKI stage (additional Table 6, additional Figs. 1 and 2) although small numbers for the birth cohort precluded in depth analysis.

## Discussion

This study examined the correlation between AKI, as identified using electronic alerts based on a relative rise in serum creatinine levels, and clinical coding within the electronic hospital record for a national cohort of children. Overall, sensitivity of coding within the hospital record was poor (19.7%), increasing with peak AKI severity. Higher odds of being coded for AKI within the electronic record were seen with older age, while being in the birth cohort conferred lower likelihood of coding, particularly for preterm infants. Children living in less deprived quintiles (quintiles 1 and 3) had higher odds of coding compared to those living in the most deprived quintile. No correlation was noted between proportion of AKI alert-episodes coded and 30-day mortality on a hospital level.

With the potential to improve AKI recognition and care, many healthcare providers, including the National Health Service, have now implemented clinical indicator alerts as part of routine practice [10–13]. Most research seeking to validate these systems are based on adult cohorts, using administrative records as the reference standard; UK-based studies demonstrate that clinical coding in the hospital record has low sensitivity for AKI detected using the ‘Think Kidneys’ e-alert algorithm, which improves with higher peak AKI stage [7, 14]. Systematic review findings support these observations among adult populations, demonstrating higher sensitivity of coding among patients with evidence of more severe AKI, irrespective of definition used. Specificity for coded AKI is, however, consistently high, indicating that while the incidence of AKI using administrative codes may be underestimated, the risk of misclassification is low [15]. Less is known, however, about the usefulness of e-alerts in the clinical recognition of AKI in children. This study, which benefits from its large sample size and data capture on a national level, found that the sensitivity of electronic hospital record coding for AKI e-alerts in children was much lower at any peak AKI stage than previously reported among adult patients [7], suggesting that, in its current form, alerts from the NHS England algorithm do not equate to clinical recognition of AKI in children. Overall, administrative coding of AKI identified using relative changes in serum creatinine (19.7%) is lower than previously reported among other studies with multi-centre coverage [16, 17] but higher than that reported for critically ill children [18]. As seen by D’Arienzo and colleagues, higher rates of coding were noted for moderate- to severe-AKI episodes, suggesting that markedly abnormal creatinine values compared to baseline are more likely to be recognised and coded as AKI. This is supported by clinical audit findings that cases of clinically recognised AKI tend to have a higher median serum creatinine value than those that are not [17].

AKI is associated with later adverse outcomes such as hypertension, proteinuria, and chronic kidney disease, for which there are treatments that can help reduce disease progression. As children stand to gain important long-term benefits from early detection, understanding how e-alerts can be used to facilitate AKI recognition is urgently needed. Among adult patients, e-alerts are variably implemented and rarely incorporate instructions for clinical care [11], however systematic review findings suggest they could modify processes of care, particularly when intermediate steps are incorporated [11]. This potential has been demonstrated among paediatric cohorts: Goldstein and colleagues successfully demonstrated a 64% reduction in AKI incidence and 38% decrease in nephrotoxin exposure for ‘at-risk’ children when an e-alert prompted follow-up blood test monitoring, suggesting that e-alerts, in conjunction with active management, may improve AKI rates [19]. At hospital level, marked variation in the percentage of AKI episodes coded in the clinical record was noted, which may be due in part to how alerts are implemented clinically at individual hospitals and whether there are services or personnel available to support to AKI recognition and management. Understanding differences in clinical and coding practices for hospitals with high and low coding rates is required to drive quality improvements in clinical care, which will aid future disease surveillance.

Patient characteristics associated with lower likelihood of AKI coding in adults include younger age, female sex and South Asian or Black ethnicity [7]. Within the paediatric population, this study demonstrated a similar relationship between age and likelihood of coding; we hypothesise this graded association may be related to perceived risk of AKI by age, which may be confounded by factors such as co-existing disease and polypharmacy. The observation that among adults, elderly patients with multiple comorbidities are more likely to be coded for AKI in the medical record [20] raises the suggestion that clinicians are predisposed to consider AKI in traditionally ‘high risk’ patients, with multiple and/or complex medical conditions who may be on a number of medications; in contrast, as the majority of children attending hospital do not have complex or chronic conditions [21, 22], they may perceived to be at lower risk. An alternative hypothesis is that smaller children have inherently lower creatinine values and therefore the relatively small changes which flag an alert may be considered insignificant and/or overlooked. This is supported by this study’s finding that children experiencing AKI episodes during their birth admission were less likely to be coded for AKI, and by a US study which found patients with AKI were younger, smaller for age and had lower baseline creatinine values compared to those without [23]. As younger children, in particular neonates, are at high risk of adverse outcomes associated with AKI, including prolonged length of stay and mortality [5, 24], it is imperative factors mediating this association are understood and addressed.

Compared to the UK general population [25], children of non-White ethnicity were over-represented in our study sample, however this fails to consider the ethnic distribution of the exposed hospitalised cohort, the data for which were unavailable. This study also noted a higher burden of AKI among children living in more socioeconomically deprived areas, which has been described for children in Wales; in the US, higher AKI rates are reported among children without medical insurance [26, 27]. Socioeconomic deprivation is associated with higher age-adjusted mortality in adults, which is due to more severe AKI and a higher prevalence of pre-existing kidney disease [28, 29]. This study has found some evidence to suggest that children from more socioeconomically deprived areas were less likely to be coded for AKI compared to children living in areas of relative affluence. This observation raises the possibility of clinical bias in AKI recognition and/or coding, which warrants further investigation. Whether this association correlates with worse outcomes also requires review.

We failed to observe a correlation between clinical coding and 30-day mortality at a hospital level, which is in contrast to studies examining the association at patient-level [18]. As more severe cases of kidney disease, both acute and chronic, tend to be reported within the electronic health record [15, 30–32], the lack of association is encouraging. This result is a crude observation however and therefore must be interpreted cautiously. In a post hoc analysis, we noted children coded for AKI experienced generally higher lengths of stay in hospital than those who were not coded; as has been demonstrated in the adult CKD literature [32], this finding may suggest children who are more severely unwell (and thus have a longer length of stay) are captured more readily than those with milder disease.

This study examined the sensitivity of hospital records to reflect AKI episodes as identified using an electronic AKI alert algorithm which is standardised and implemented across NHS hospitals nationally, therefore it is anticipated these results will be generalisable to devolved UK nations. In their current form, electronic hospital records are likely to underestimate the incidence of AKI in children. This study has demonstrated the value of e-alerts in identifying where gaps occur in AKI coding, as a proxy for clinical recognition, and factors associated with coding in the clinical record. In the NHS, laboratory AKI alerts will not necessarily translate into the patient record without clinician recognition and documentation of AKI as a diagnosis. Further work is now needed to understand the reasons for these associations and to reduce potential inequalities in recognition which may have clinical implications, for management both acutely and in the long-term, given the growing evidence of the long-term consequences of AKI [33–35].

However, this study has limitations. Not all laboratories across England submitted data in time for linkage to HES; we estimate this report represents data from 66% of NHS laboratories across England [5]. Analyses were restricted to clinical codes for AKI using the N17.x code, which may have missed cases classified using procedural (kidney replacement therapy) or other diagnostic ICD-10 codes, for example N19 (unspecified kidney failure). Additionally, the presence of an AKI code (yes/no) was determined by a diagnostic code present at any time during an AKI-associated hospitalisation; given that corresponding diagnostic codes may be entered later in the electronic record, this may again underestimate the validity of the electronic record for detecting episodes derived from AKI alert data [15]. This study defined AKI using a national algorithm which uses relative changes in serum creatinine, which in children may inadvertently misclassify spurious values; this method may also miss cases of AKI using alternative measures, such as urine output [36]. There are now validated studies which impute baseline serum creatinine values which may support AKI identification in children who have not previously had a kidney function test performed [37, 38].

Finally, to what extent alerts truly reflect AKI, particularly stage one, requires further evaluation. While a standardised algorithm is used nationally, it is our understanding that locally, units may suppress alerts for certain populations (e.g., neonates, children known to kidney teams) therefore to what extent this influences clinical recognition (and therefore management) also requires review.

In conclusion, coding in hospital records shows poor agreement with AKI episodes defined using a rise in serum creatinine from baseline for children and young people, suggesting a lack of clinical recognition. Factors associated with lower levels of coding included lower stages of AKI, younger age, experiencing an AKI episode around the time of birth and living in a more deprived area. At hospital-level, the presence of AKI coding, as a proxy measure for recognition, did not correlate with 30-day mortality. Further work is now required to understand how e-alerts can be used to improve clinical recognition of AKI in children, to enhance care and outcomes.

## Supplementary Information

Supplementary Material 1.

Supplementary Material 2.

Supplementary Material 3.

## Data Availability

The data underlying this article were provided by the UK Renal Registry (AKI data) and NHS Digital (Hospital Episode Statistics data) under license/by permission and are not publicly available. Data will be shared on request to the corresponding author with permission of the UK Renal Registry and NHS Digital.

## Abbreviations

AKI: Acute Kidney Injury
CA: Community-acquired acute kidney injury
e-: Electronic
HA: Hospital-acquired acute kidney injury
HES: Hospital Episode Statistics
ICD-10: International Classification of Diseases, tenth revision
NHS: National Health Service
UKRR: UK Renal Registry
