# Supplementary material for: Identifying acute kidney injury in children: comparing electronic alerts with health record data
Source: BMC Nephrol. 2025 Feb 13;26:75. doi: 10.1186/s12882-025-03961-3 (PMC11827200; doi:10.1186/s12882-025-03961-3)
Supplement: Supplementary file 1 — Supplementary Material 1. [file 12882_2025_3961_MOESM1_ESM.docx]

**Identifying acute kidney injury in children: comparing electronic alerts with health record data.**

Lucy Plumb^1,2^, Manuela Savino^1^, Anna Casula^1^, Manish D Sinha^3,4^, Carol D Inward^5^, Stephen D Marks^6,7^, James Medcalf^1,8,9^, Dorothea Nitsch^1,10^

**Additional table 1: OPCS procedure codes used to identify children in receipt of kidney replacement therapy.**

| **OPCS code** | **Description** |
| --- | --- |
| X40 | Dialysis NEC |
| X401 | Renal dialysis |
| X402 | Peritoneal dialysis NEC |
| X403 | Haemodialysis NEC |
| X405 | Automated peritoneal dialysis |
| X406 | Continuous ambulatory peritoneal dialysis |
| X408 | Other specified compensation for renal failure |
| X409 | Unspecified compensation for renal failure |

**Additional table 2: Hospitals submitting e-alert 2017 data to UK Renal Registry included in analysis**

Basildon And Thurrock University Hospitals NHS Foundation Trust

East Kent Hospitals University NHS Foundation Trust

Great Western Hospitals NHS Foundation Trust

Kettering General Hospital NHS Foundation Trust

North Middlesex University Hospital NHS Trust

The Royal Wolverhampton NHS Trust

Gloucestershire Hospitals NHS Foundation Trust

Plymouth Hospitals NHS Trust

University Hospitals Coventry and Warwickshire NHS Trust

Bradford Teaching Hospitals NHS Foundation Trust

Taunton And Somerset NHS Foundation Trust

Stockport NHS Foundation Trust

Calderdale And Huddersfield NHS Foundation Trust

Royal Cornwall Hospitals NHS Trust

Barts Health NHS Trust

University Hospitals of North Midlands NHS Trust

University Hospitals of Leicester NHS Trust

University College London Hospitals NHS Foundation Trust

The Newcastle Upon Tyne Hospitals NHS Foundation Trust

Medway NHS Foundation Trust

Great Ormond Street Hospital for Children NHS Foundation Trust*

Portsmouth Hospitals NHS Trust

Nottingham University Hospitals NHS Trust

Sheffield Children's NHS Foundation Trust*

King's College Hospital NHS Foundation Trust

University Hospitals Bristol NHS Foundation Trust

Leeds Teaching Hospitals NHS Trust

University Hospital Southampton NHS Foundation Trust

Alder Hey Children's NHS Foundation Trust*

City Hospitals Sunderland NHS Foundation Trust

The Dudley Group NHS Foundation Trust

East Sussex Healthcare NHS Trust

Royal Surrey County Hospital NHS Foundation Trust

University Hospitals of Morecambe Bay NHS Foundation Trust

United Lincolnshire Hospitals NHS Trust

Shrewsbury And Telford Hospital NHS Trust

Manchester University NHS Foundation Trust

University Hospitals Birmingham NHS Foundation Trust

Epsom And St Helier University Hospitals NHS Trust

The Queen Elizabeth Hospital, King's Lynn, NHS Foundation Trust

Barking, Havering and Redbridge University Hospitals NHS Trust

London North West University Healthcare NHS Trust

Yeovil District Hospital NHS Foundation Trust

Southend University Hospital NHS Foundation Trust

Dartford And Gravesham NHS Trust

Doncaster And Bassetlaw Teaching Hospitals NHS Foundation Trust

Derby Teaching Hospitals NHS Foundation Trust

Lancashire Teaching Hospitals NHS Foundation Trust

Poole Hospital NHS Foundation Trust

Hull And East Yorkshire Hospitals NHS Trust

Mid Yorkshire Hospitals NHS Trust

Royal United Hospitals Bath NHS Foundation Trust

Milton Keynes University Hospital NHS Foundation Trust

Ashford And St Peter's Hospitals NHS Foundation Trust

Surrey And Sussex Healthcare NHS Trust

Worcestershire Acute Hospitals NHS Trust

St Helens And Knowsley Hospital Services NHS Trust

York Teaching Hospital NHS Foundation Trust

Frimley Health NHS Foundation Trust

Northern Lincolnshire and Goole NHS Foundation Trust

Norfolk And Norwich University Hospitals NHS Foundation Trust

Tameside And Glossop Integrated Care NHS Foundation Trust

East Lancashire Hospitals NHS Trust

Northern Devon Healthcare NHS Trust

Ipswich Hospital NHS Trust

Bolton NHS Foundation Trust

Hampshire Hospitals NHS Foundation Trust

*Hospital trusts providing paediatric services only

**Additional table 3: Coding of AKI by prematurity status for birth cohort.**

| **Birth type** | **Episodes by peak AKI stage** | | |
| --- | --- | --- | --- |
|  | **1** | **2** | **3** |
| Birth total cohort N | 384 | 206 | 142 |
| % coded as AKI (N17) | 4.7 | 7.3 | 21.1 |
| Non preterm birth N | 106 | 62 | 45 |
| % coded as AKI (N17) | 9.4 | 8.1 | 28.9 |
| Preterm birth N | 278 | 144 | 97 |
| % coded as AKI (N17) | 2.9 | 6.9 | 17.5 |

*Abbreviations: AKI, Acute Kidney Injury.*

**Additional table 4: Coding of AKI by critical care admission (yes/no) and peak AKI stage**

| **Peak AKI stage** | **Coded for AKI (N17)** | **Critical care admission** | |
| --- | --- | --- | --- |
|  |  | **No (%)** | **Yes (%)** |
| **1** | **Yes** | 469 (14.0) | 131 (16.8) |
|  | **No** | 2881 (86.0) | 647 (83.2) |
| **2** | **Yes** | 202 (21.1) | 93 (25.5) |
|  | **No** | 754 (78.9) | 272 (74.5) |
| **3** | **Yes** | 224 (39.7) | 117 (45.2) |
|  | **No** | 340 (60.3) | 142 (54.8) |

*Abbreviations: AKI, Acute Kidney Injury.*

**Additional table 5: Coding of AKI by critical care admission (yes/no) and peak AKI stage**

| **Peak AKI stage** | **Coded for AKI (N17)** | **KRT (dialysis) use** | |
| --- | --- | --- | --- |
|  |  | **No (%)** | **Yes (%)** |
| **1** | **Yes** | 587 (14.4) | 13 (34.2) |
|  | **No** | 3503 (85.7) | 25 (65.8) |
| **2** | **Yes** | 290 (22.3) | 5 (25.0) |
|  | **No** | 1011 (77.7) | 15 (75.0) |
| **3** | **Yes** | 290 (38.9) | 51 (66.2) |
|  | **No** | 456 (61.1) | 26 (33.8) |

*Abbreviations: AKI, Acute Kidney Injury; KRT, Kidney Replacement Therapy*

**Additional table 6: Median length of stay by AKI type and peak AKI stage.**

| **AKI type** | **Peak AKI stage** | **Coded for AKI** | **N** | **Median length of stay (IQR)** |
| --- | --- | --- | --- | --- |
| Preterm birth | 1 | No | 270 | 43 (22, 72) |
|  |  | Yes | 8 | 62 (24, 76) |
|  | 2 | No | 134 | 35 (21, 74) |
|  |  | Yes | 10 | 69 (58, 87) |
|  | 3 | No | 80 | 51 (26, 80) |
|  |  | Yes | 17 | 45 (33, 80) |
| Non-preterm birth | 1 | No | 96 | 23 (13, 46) |
|  |  | Yes | 10 | 87 (18, 180) |
|  | 2 | No | 57 | 24 (15, 41) |
|  |  | Yes | 5 | 22 (15, 46) |
|  | 3 | No | 32 | 26 (12, 72) |
|  |  | Yes | 13 | 39 (25, 56) |
| Community acquired and hospitalised AKI | 1 | No | 1705 | 3 (2, 7) |
|  |  | Yes | 282 | 5 (2, 11) |
|  | 2 | No | 427 | 5 (3, 10) |
|  |  | Yes | 127 | 7 (4, 13) |
|  | 3 | No | 210 | 5 (3, 15) |
|  |  | Yes | 190 | 10 (5, 24) |
| Hospital acquired AKI | 1 | No | 1457 | 13 (7, 29) |
|  |  | Yes | 300 | 14 (8, 30) |
|  | 2 | No | 408 | 22 (10, 47) |
|  |  | Yes | 153 | 25 (12, 64) |
|  | 3 | No | 169 | 21 (9, 43) |
|  |  | Yes | 121 | 25 (13, 54) |

*Abbreviations: AKI, Acute Kidney Injury; IQR, Interquartile range.*
