# Supplementary material for: Identifying acute kidney injury in children: comparing electronic alerts with health record data
Source: BMC Nephrol. 2025 Feb 13;26:75. doi: 10.1186/s12882-025-03961-3 (PMC11827200; doi:10.1186/s12882-025-03961-3)
Supplement: Supplementary file 3 — Supplementary Material 3. [file 12882_2025_3961_MOESM3_ESM.pdf]

Additional Figure 2: Length of stay for birth cohort, stratified by AKI type.

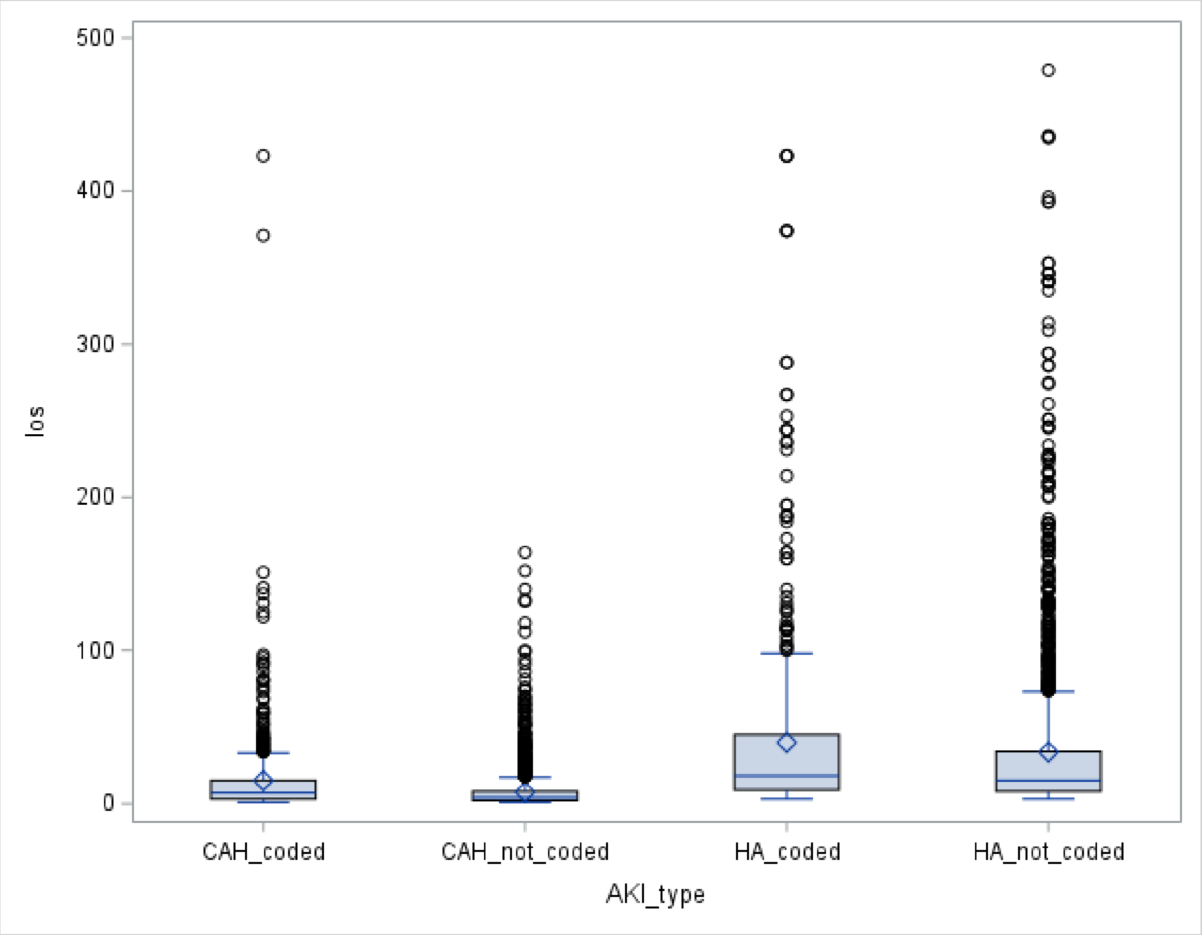

Abbreviations: AKI, Acute Kidney Injury; CAH, community acquired and hospitalised; HA, hospital acquired; LOS, Length of stay.
